# A discovery down under: decoding the draft genome sequence of *Pantoea stewartii* from Australia’s Critically Endangered western ground parrot/kyloring (*Pezoporus flaviventris*)

**DOI:** 10.1099/mgen.0.001101

**Published:** 2023-09-04

**Authors:** Rhys T. White, William Taylor, Natalie Klukowski, Rebecca Vaughan-Higgins, Ernest Williams, Steve Petrovski, Jayson J. A. Rose, Subir Sarker

**Affiliations:** ^1^​ Institute of Environmental Science and Research, Wellington, New Zealand; ^2^​ Institute of Environmental Science and Research, Christchurch, New Zealand; ^3^​ Department of Microbiology, Anatomy, Physiology and Pharmacology, School of Agriculture, Biomedicine and Environment, La Trobe University, Melbourne, Victoria, Australia; ^4^​ Perth Zoo, South Perth, Western Australia, Australia; ^5^​ Institute of Environmental Science and Research, Wallaceville, New Zealand; ^6^​ Biomedical Sciences & Molecular Biology, College of Public Health, Medical and Veterinary Sciences, James Cook University, Townsville, QLD, Australia

**Keywords:** Australia, birds, *Enterobacterales*, *Pantoea stewartii* subsp. *indologenes*, phylogenomics, strain C10109_Jinnung

## Abstract

*
Pantoea stewartii *, a plant pathogen, is primarily transmitted through contaminated seeds and insect vectors, with the corn flea beetle (*Chaetocnema pulicaria*) being the primary carrier. *P. stewartii* is a bacterium belonging to the order *Enterobacterales* and can lead to crop diseases that have a significant economic impact worldwide. Due to its high potential for spread, *P. stewartii* is classified as a quarantine organism in numerous countries. Despite its impact on agriculture, the limited genome sequences of *P. stewartii* hamper understanding of its pathogenicity and host specificity, and the development of effective control strategies. In this study, a *P. stewartii* strain (C10109_Jinnung) was discovered in the faecal matter of the Critically Endangered western ground parrot/kyloring (*Pezoporus flaviventris*) in Australia, which to our knowledge is the first reported *P. stewartii* genome from a bird source. Whole-genome sequencing and phylogenomic analysis of strain C10109_Jinnung, obtained from a captive psittacine, provides new insights into the genetic diversity and potential transmission route for the spread of *P. stewartii* beyond insects and plants, where *P. stewartii* is typically studied. Our findings provide new insights into the potential transmission route for spread of *P. stewartii* and expand the known transmission agents beyond insects and plants. Expanding the catalogue of *P. stewartii* genomes is fundamental to improving understanding of the pathogenicity, evolution and dissemination, and to develop effective control strategies to reduce the substantial economic losses associated with *P. stewartii* in various crops and the potential impact of endangered animal species.

## Data Summary

The study sequences are available in the National Center for Biotechnology Information (NCBI) under BioProject accession number PRJNA967810. The raw Illumina sequence read data generated in this study have been deposited at the NCBI sequence read archive [SRA (https://www.ncbi.nlm.nih.gov/sra)] under accession number SRR24455997. The draft assembly has been deposited in GenBank under accession number JASCYM000000000. The software used to analyse raw sequence reads for polymorphism discovery and whole-genome sequencing-based phylogenetic reconstruction are available as described in the Methods. The authors confirm that all supporting data protocols have been provided in the article or supplementary data files.

**Impact Statement**

This study reports the draft genome sequence of a *Pantoea stewartii* strain, C10109_Jinnung, found in the faecal matter of a Critically Endangered bird species, the western ground parrot/kyloring (*Pezoporus flaviventris*), in Australia. Genome-wide taxonomic analysis showed that *P. stewartii* genome CCUG 26359 was the nearest taxonomic neighbour to C10109_Jinnung within the *P. stewartii* population. Phylogenomic analyses revealed that C10109_Jinnung was most genomically similar to the *P. stewartii* subsp. *indologenes* genome ZJ-FGZX1, isolated from lucky bamboo (*Dracaena sanderiana*) in China. However, a substantial genomic divergence was indicated by a distance of 18 697 pairwise single-nucleotide variants between the genomes of ZJ-FGZX1 and C10109_Jinnung. These findings expand the known transmission pathways for the spread of *P. stewartii* beyond insects and plants to birds, which may have important implications for developing effective control strategies. The results of this study provide new insights into the genetic diversity of *P. stewartii* and highlight the need for further genomic studies to better understand the molecular basis of pathogenicity, evolution and dissemination of this important plant pathogen.

## Introduction

*Pantoea stewartii* (syn. *Erwinia stewartii *) is a Gram-negative bacterium that causes Stewart’s wilt (young seedlings) and leaf blight (mature plants) of economically important crops such as jackfruit (*Artocarpus heterophyllus*) [1], rice (*Oryza sativa*) [2], sudangrass (*Sorghum drummondii*) [3] and corn (*Zea mays*) [4]. In particular, the corn industry has suffered substantial economic losses due to the impact of *P. stewartii* [5–7]. For instance, in the USA in 1999, the state of New York alone reported losses exceeding USD$1 million in corn yield attributed to Stewart’s wilt [8]. Consequently, many countries classify *P. stewartii* as a quarantine organism [9].

*P. stewartii* can spread through contaminated seeds and insect vectors. *P. stewartii* has been associated with gut colonization and pathogenicity in various insect hosts such as the corn flea beetle (*Chaetocnema pulicaria*) [10], pea aphid (*Acyrthosiphon pisum*) [11] and yellow fever mosquito (*Aedes aegypti*) [12]. The corn flea beetle is the primary vector for *P. stewartii* [13], the spread of *P. stewartii* between plants depends on the presence of this beetle, and the incidence of Stewart’s wilt/leaf blight is directly related to the density of the corn flea beetle population in corn fields [10, 14, 15]. Although *P. stewartii* substantially impacts agriculture, there is limited information on its genome sequence. Only 29 draft and three complete genome sequences have been deposited in the public National Center for Biotechnology Information (NCBI) databases (as of March 2023), including the complete genome sequences *P. stewartii* subsp. *indologenes* strain ZJ-FGZX1 [16], *P. stewartii* subsp. *stewartii* strain DC283 [17] and *P. stewartii* strain HR3-48 [18]. Therefore, expanding the catalogue of *P. stewartii* genomes is fundamental for an improved understanding of the genetic and molecular basis of pathogenicity, evolution, dissemination and potential host specificity, and for developing effective control strategies.

The main culprit responsible for causing Stewart’s wilt of corn is *P. stewartii* subsp. *stewartii *, as opposed to *P. stewartii* subsp. *indologenes* [19–21]. Despite the relatively few known genotypic differences between these subspecies, they can still be distinguished based on variations in ‘electrophoretic protein profiles, fatty acid composition, and biochemical properties’ [19, 22]. Furthermore, specific single-nucleotide variants (SNVs) identified in housekeeping genes, such as *recA* (encoding protein RecA) and *galE* (encoding UDP-glucose 4-epimerase), provide additional markers for differentiation [20, 21]. Nevertheless, these analyses are not always completely reliable in distinguishing between different species of *Pantoea* or subspecies of *P. stewartii* [20]. However, by exploring and comparing the genomes of different *P. stewartii* subspecies, researchers can gain valuable insights into their distinct characteristics and virulence factors.

*P. stewartii* has yet to be documented in Australia. However, there is a considerable risk of its introduction through maize grains imported from the USA. Given that Australia has extensive maize cultivation and suitable environmental conditions for Stewart’s wilt disease, there is potential for substantial yield losses. Therefore, the entry of *P. stewartii* poses a quarantine risk to Australia. The extent of this risk depends on evaluating local insects as potential vectors of *P. stewartii *, making quantification challenging. While investigating unknown circulating pathogens, *P. stewartii* was discovered in the faecal matter of the Critically Endangered western ground parrot/kyloring (*Pezoporus flaviventris*). To the best of our knowledge, there are no publicly available genomic studies of *P. stewartii* genomes from birds in Australia, which raises questions about the genetic diversity of circulating strains and their potential transmission pathways. This finding was unexpected but not unprecedented, as a similar discovery was made with *Pantoea eucalypti* in goose (*Branta canadensis*) faeces from Canada [23]. Due to the novelty of discovering *P. stewartii* from an avian source, we conducted whole-genome sequencing and phylogenomic analyses on a *P. stewartii* strain obtained from a captive psittacine in Australia. This allowed us to place the strain within the larger global phylogenetic context of *P. stewartii *. By employing metagenomic analyses, our study has yielded valuable insights into the potential mechanisms underlying the dissemination of *P. stewartii *. While our study revealed the presence of *P. stewartii* in bird faeces, seeds may serve as a more probable reservoir or source of infection for the spread of *P. stewartii *, rather than the bird itself acting as the primary host. These findings broaden the spectrum of known transmission routes associated with *P. stewartii* beyond the conventional focus on insects and plants, thus enhancing our understanding of its transmission dynamics.

## Methods

### Clinical history

The clinical history provided pertains to a male western ground parrot/kyloring captured in April 2021 as part of a translocation programme in Western Australia. The capture and subsequent transport to camp were uneventful, but a single damaged secondary feather was noted on the right wing. As only one feather was affected, the veterinarian interpreted the damage as follicular rather than feather disease. After several unsuccessful attempts to fly, the bird was deemed unfit for release to the wild and transported to Perth Zoo (South Perth, Western Australia) for further assessment and care. Upon arrival at Perth Zoo, the parrot was observed to have a right-wing droop and a small alopecic area on the crown of his head where feather loss was evident, possibly from trauma during transport. The bird tested negative for psittacine beak and feather disease (PBFD) virus, chlamydia, polyoma virus, psittacine herpesvirus and parasites in May 2021. The bird had lost 9 % of its body weight since capture, probably reflecting a combination of true weight loss, dehydration and a smaller amount of seed (native food plants with other grains) being offered (Fig. S1, available in the online version of this article). The bird’s diet consisted of either a zoo mix or imported parrot pellets from the USA (which may have contained corn). The veterinarians believed the right-wing droop indicated a musculoskeletal injury and treated the western ground parrot/kyloring with the anti-inflammatory medication meloxicam at 0.5 mg kg^–1^ via intramuscular injection. Later, the bird was observed drinking from a water bowl and eating seeds from a tray. However, the right-wing droop was still intermittently evident.

### Sampling, ethical considerations and extraction of DNA

Faecal samples were obtained from the western ground parrot/kyloring declared unfit for translocation at Perth Zoo in 2021. To the best of our knowledge, there are no other samples in the world from this species/sample type as it is endemic to Western Australia, exceedingly rare, and critically endangered. Faecal samples were obtained during routine animal husbandry practice with no handling or other intervention required. The psittacine was housed individually in the aviary, and the faecal sample was collected from the temporary holding box (Fig. S1). The attending veterinarian collected the faecal samples and shipped samples to La Trobe University (Melbourne, Victoria, Australia) to investigate circulating unknown pathogens. The Animal Ethics Committee at La Trobe University was informed that findings from the material (with no bird intervention) were to be used in a publication, and a formal waiver of ethics approval was granted. Faecal materials were aseptically resuspended and homogenized vigorously in sterile PBS (1 : 10), and the DNA was extracted as per previous methods [24, 25] using a ReliaPrep gDNA Tissue Miniprep System (Promega).

### Library construction and next-generation sequencing

A total of 7 ng of extracted genomic DNA (from the faecal materials) was used to prepare the library using the protocol adapted previously using the Illumina DNA Prep (Illumina) for Illumina short-read sequencing [26]. The quality and quantity of the prepared library were assessed using an Agilent Tape Station (Agilent Technologies) by the Australian Genome Research Facility (AGRF) (Parkville, Victoria, Australia). The prepared library was sequenced at the AGRF, producing 2×150 bp paired-end reads using the NovaSeq 6000 platform, according to the manufacturer’s instructions. Subsequently, an attempt was made to prepare a long-read library using the SQK-LSK109 ligation sequencing kit with native barcoding expansion EXP-NBD104 (Oxford Nanopore Technologies). Regrettably, we encountered difficulties in generating a successful library due to the limited quantity of input DNA available. The protocol was further modified to accommodate a low gDNA input (approximately 53.0 ng) using half reaction volumes as per the stated protocol [27]. The final DNA yield in the prepared long-read library was too low to detect when using a Qubit 3.0 Fluorometer (Thermo Fisher Scientific), and thus analyses were not progressed.

### Quality trimming and *de novo* assembly of the sequence read data

Raw reads were checked for quality using FastQC v0.11.9 (http://www.bioinformatics.babraham.ac.uk/projects/fastqc/, accessed on 10 March 2023). Raw sequence reads were *de novo* assembled using Shovill v1.1.0 (https://github.com/tseemann/shovill, accessed on 10 March 2023), which utilizes: Seqtk v1.3-r106 (https://github.com/lh3/seqtk, accessed on 10 March 2023); Trimmomatic v0.36 [28]; Lighter v1.1.2 [29]; FLASH v1.2.11 [30]; SKESA v2.4.0 [31, 32]; Samclip v0.4.0 (https://github.com/tseemann/samclip, accessed on 10 March 2023); SAMtools v1.16.1 [33], Burrows-Wheeler Aligner (BWA) v0.7.17 [34]; and Pilon v1.24 [35]. Shovill was used with parameters set to: (i) estimate the genome size to 5 Mb; (ii) remove contiguous sequences (contigs) with a sequence coverage below 20-fold; and (iii) enable single-cell mode. Assembly metrics were assessed using QUAST v5.0.2 [36]. A genome sequencing coverage plot was created with the script ‘wgscoverageplotter.jar’ from the Java utilities for Bioinformatics (JVARKIT) (https://github.com/lindenb/jvarkit).

### *In silico* species identification and antibiotic resistance genotyping

To perform taxonomic profiling and identify *P. stewartii* by classifying the trimmed paired-end DNA sequences, we used Kraken v2.1.3 [37] with default parameters and an NCBI Reference Sequence (RefSeq) database [38], PlusPFP (https://benlangmead.github.io/aws-indexes/k2). The database contained references for archaea, bacteria, human, viruses, plasmids, protozoa, fungi, plant, and the ‘UniVec core’ subset of the UniVec database (a database of vector, adaptor, linker and primer sequences). Full taxonomic profiles of paired-end reads can be found in Table S1. ABRicate v1.0.1 (https://github.com/tseemann/abricate, accessed on 13 March 2023) was used to screen the draft assembly for acquired antibiotic resistance genes using the ARG-ANNOT [39] database (last updated 1 November 2022). The genome sequence for a putative plasmid (named pC10109_Jinnung) was annotated using RAST v2.0 [40–42]. The genome map for pC10109_Jinnung was visualized with MacVector v16.0 (https://macvector.com).

### Taxonomic classification of the C10109_Jinnung genome

To identify the nearest taxonomic neighbours to the genome of C10109_Jinnung, the draft assembly was input into a Genome Taxonomy Database Toolkit (GTDB-Tk) genome-based taxonomy (GTDB-Tk v2.1.1 with GTDB package R207_v2 [43, 44]). Only sequences within the genus *Pantoea* (g_, genus) were extracted from the GTDB-Tk and used to construct a concatenated reference alignment of 119 bacterial marker genes. The taxonomic tree was inferred using maximum-likelihood approximation with FastTree v2.1.7 [45] under the WAG model [46] of protein evolution with gamma-distributed rate heterogeneity [47] (+GAMMA).

### Variant detection and phylogenetic analyses

To estimate the number of SNVs and phylogenetic relationships between the genome of C10109_Jinnung and other *P. stewartii* genomes sequenced elsewhere, these were compared to a global and publicly available dataset (Table S2). Parsnp v1.7.4 [48] was used to generate a core-genome alignment (alignment of the syntenic regions across all genomes). The resulting SNV alignments were used to reconstruct phylogenies. Maximum-parsimony trees were reconstructed using the heuristic search feature of PAUP v4.0a [49], before adding 1000 bootstrap replicates. The resulting phylogenetic trees were visualized using FigTree v1.4.4 (http://tree.bio.ed.ac.uk/software/figtree/, accessed on 10 March 2023).

### Metagenomic analysis

Metagenomic data assemblies were produced using SPAdes v3.15.5 (with the --meta flag) [50], and the identification of taxa based on paired-end reads and contigs was performed using Kraken v2.1.3 and the aforementioned PlusPFP database. Additionally, identified contigs were extracted using KrakenTools [51] and subjected to further investigation through alignment using BWA v0.7.15 and SAMtools v1.15.1. For metagenomic taxonomy investigation and analysis, R v4.0.5 [52] was utilized along with the phyloseq v1.34.0 [53] and Pavian v1.2.0 [54] packages. Trimmed reads were also aligned using BWA to the C10109_Jinnung assembly to further quantify their respective abundance, using mapQ>30 counts produced with SAMtools. Full taxonomic profiles of assembled reads can be found in Table S3.

## Results

### The genome of *P. stewartii* strain C10109_Jinnung

**Fig. 1.**
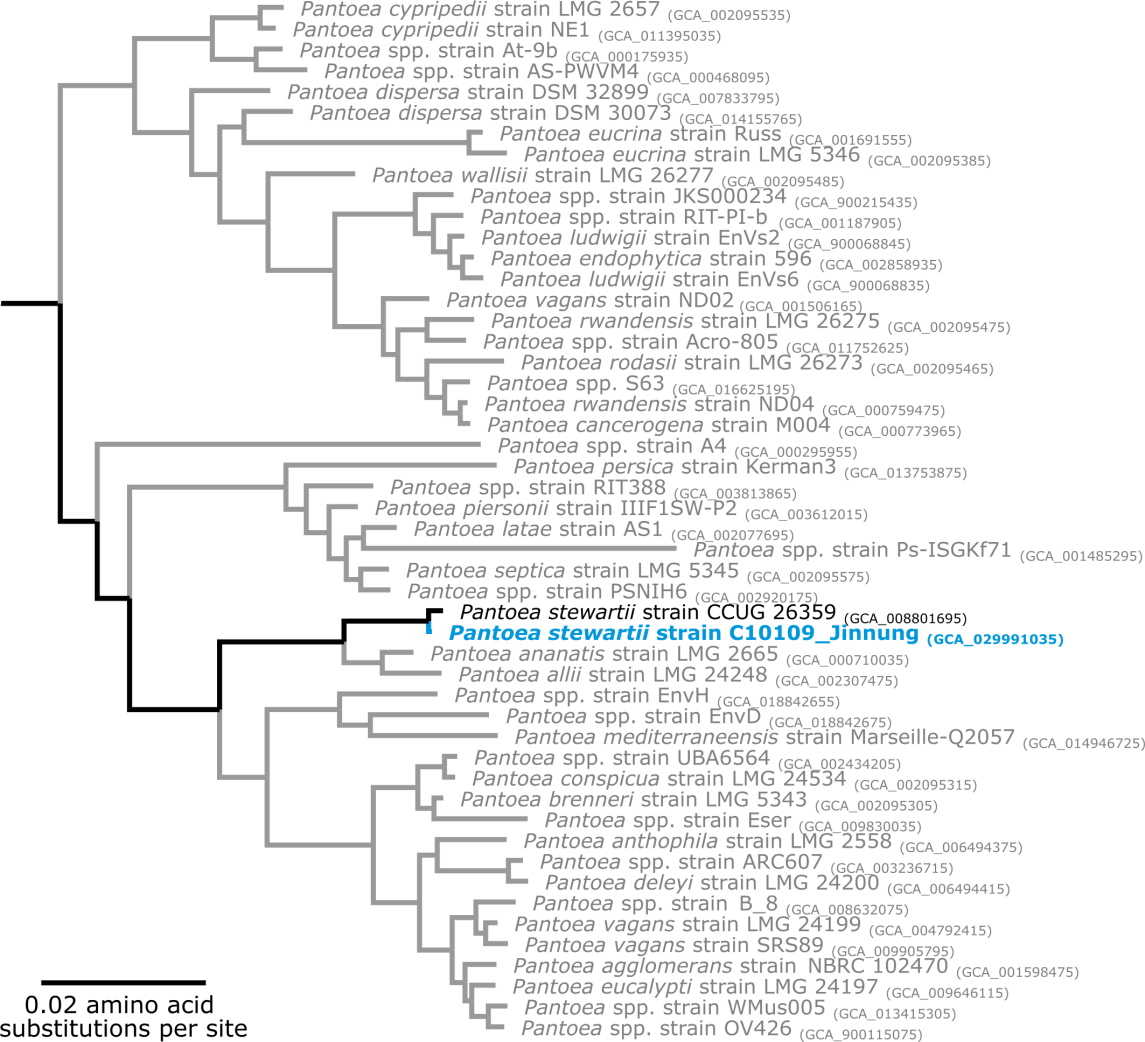
Taxonomic tree of the genus *Pantoea *. The maximum-likelihood approximation was reconstructed using a concatenated alignment of 119 conserved bacterial markers. Taxonomy is shown according to the GTDB (s_, species) from NCBI taxonomy. GenBank assembly accession numbers are displayed after the genome names. The blue taxon represents the strain sequenced in this report. The tree is rooted according to the outgroup genus *Erwinia *, which has been omitted for visualization.

The clinical metadata for the sample sequenced in this study (C10109_Jinnung) are listed in Table 1. Whole-genome sequencing resulted in the draft genome sequence of C10109_Jinnung, which comprised 4 645 191 bp (split over 20 contigs) with an average GC content of 53.72 %. Mapping the short reads from C10109_Jinnung to its draft genome shows a consistent and high median coverage exceeding 4500-fold, with a uniform distribution across the genome (i.e. a thorough representation of the genetic information in the genome of C10109_Jinnung) (Fig. S2). Table 1 also summarizes the sequence read data quality metrics for C10109_Jinnung. GTDB-Tk was used to initially classify the C10109_Jinnung strain with the nearest taxonomic neighbours within the *P. stewartii* population and verify the taxonomic assignment. Genome-wide taxonomic analysis with GTDB-Tk revealed that the C10109_Jinnung strain clusters with the draft chromosome of *P. stewartii* strain CCUG 26359 (GenBank: GCA_008801695) (Fig. 1). Fig. 1 represents a clustering analysis based on a concatenated alignment of 119 conserved genes and is therefore not a ‘go-to’ phylogenetic tree for focused clustering based on SNVs. While branches appear short in Fig. 1 they actually represent a large difference.

**Table 1.** Genome metrics and descriptions for *Pantoea stewartii* strain C10109_Jinnung

Strain characteristics	
Collection year	2021
Location	Perth, Australia
Source	*Pezoporus flaviventris*
Clinical presentation	Soft tissue injury
Anatomical site	Faecal sample
**Sequence data quality metrics**	
*Raw data*	
Average read length	150 bp
Total reads (pairs)	93 033 611
*Quality trimming*	
Average read length (range)	148 bp (36–150 bp)
Total reads (pairs) survived trimming	81 078 492 (87.1 %)
Taxonomic classification (PlusPFP database)*	
Number of unclassified reads	3 854 321 (4.8 %)
Number of reads species 1	73 662 008 (90.9 %), *Pantoea stewartii *
Number of reads species 2	849 732 (1.1 %), *Pantoea ananatis *
Number of reads species 3	196 462 (0.2 %), *Homo sapiens*
**Genomic description**	
Average genome coverage	~ 4675×
Chromosome length	4 645 191 bp
Number of contigs	20
Number of contigs >1000 bp	18
Largest contig	1 636 662 bp
GC content	53.72 %
N50	366 162 bp
Number of reads mapped to assembly (mapQ>30)	77 463 154

^∗^Full taxonomic profiles of paired-end reads can be found in Table S1.

**Fig. 2.**
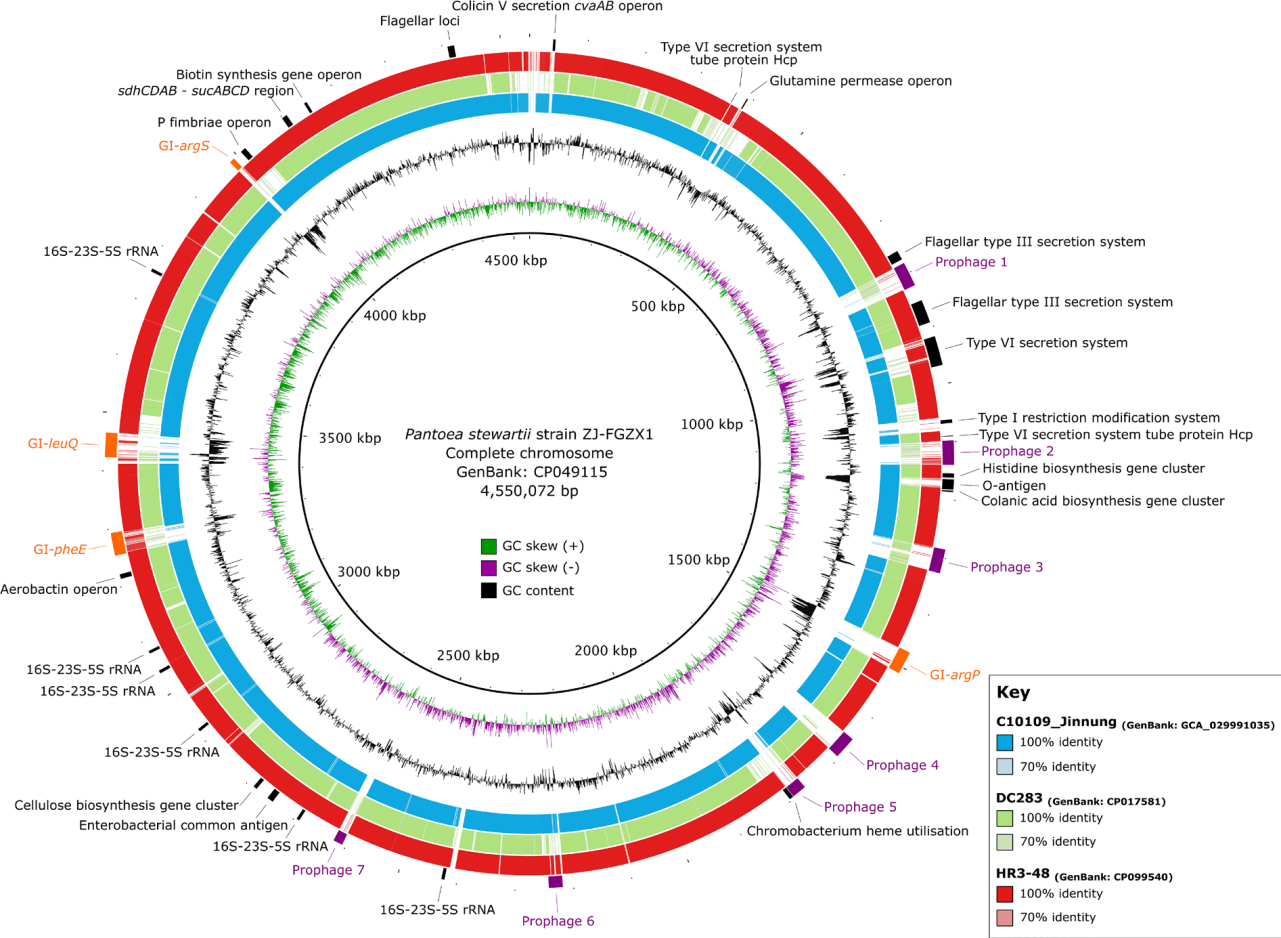
Genomic comparisons between *Pantoea stewartii *. Circular representation of the chromosome of *P. stewartii* subsp. *indologenes* strain ZJ-FGZX1 (GenBank: CP049115). The innermost rings represent the position in the genome in base pairs, GC skew and GC content. Rings 4–6 represent nucleotide identity between sequences according to BLASTn (70–100 %) between the draft genome of C10109_Jinnung (GenBank: GCA_029991035) and the complete genomes *P. stewartii* subsp. *stewartii* DC283 (GenBank: CP017581) and *P. stewartii* HR3-48 (GenBank: CP099540), as shown in the key. The outermost ring highlights regions of interest (black), genomic islands (orange) and prophages (purple). Image created using BRIG v0.95 [72].

Despite being a draft genome, the chromosome of C10109_Jinnung displayed characteristics commonly found in both *Enterobacterales* and plant-associated bacteria. Nucleotide–nucleotide blast (BLASTn) comparisons between C10109_Jinnung, the reference chromosome for strain ZJ-FGZX1 (GenBank: CP049115) and other complete *P. stewartii* genomes [DC283 (GenBank: CP017581) and HR3-48 (GenBank: CP099540)] revealed the presence of various genetic elements (Fig. 2). These included the cellulose biosynthesis gene cluster, enterobacterial common antigen, a type VI secretion system, flagella and the colanic acid biosynthesis gene cluster.

Additionally, BLASTn analysis of the draft genome of C10109_Jinnung revealed multiple regions with similarities (>70 % nucleotide identity) to genes associated with virulence relative to the complete chromosome of ZJ-FGZX1. These regions contain genes encoding: (i) the flagellum, with the extracellular components secreted by a type III secretion system, which contribute to bacterial motility; (ii) the O-antigen, a component of lipopolysaccharide linked to immune evasion; (iii) chromobacterium haem utilization and aerobactin operon, crucial for acquiring iron; and (iv) a P fimbriae operon involved in bacterial adhesion. Among the seven prophages in ZJ-FGZX1, prophage 6 was the sole prophage found to be shared with the draft genome of C10109_Jinnung (Fig. 2). In the complete chromosome of ZJ-FGZX1, prophage 6 has integrated into the tRNA-*metH* site. ZJ-FGZX1 prophage 6 comprises a total of 33 hypothetical protein-coding sequences (CDS), along with two other CDSs identified as a phage tail sheath protein (locus tag: G5574_10520) and a recombinase family protein (locus tag: G5574_10530). Caution should be exercised when interpreting the presence or similarity of ZJ-FGZX1 prophage 6 with regions in C10109_Jinnung, because the corresponding region in C10109_Jinnung is fragmented across three contigs of varying sizes: 356 933 bp (GenBank: JASCYM010000004), 1849 bp (GenBank: JASCYM010000005) and 181 569 bp (GenBank: JASCYM010000006).

### The genome of C10109_Jinnung is genomically distinct from other *P. stewartii* genomes

**Fig. 3.**
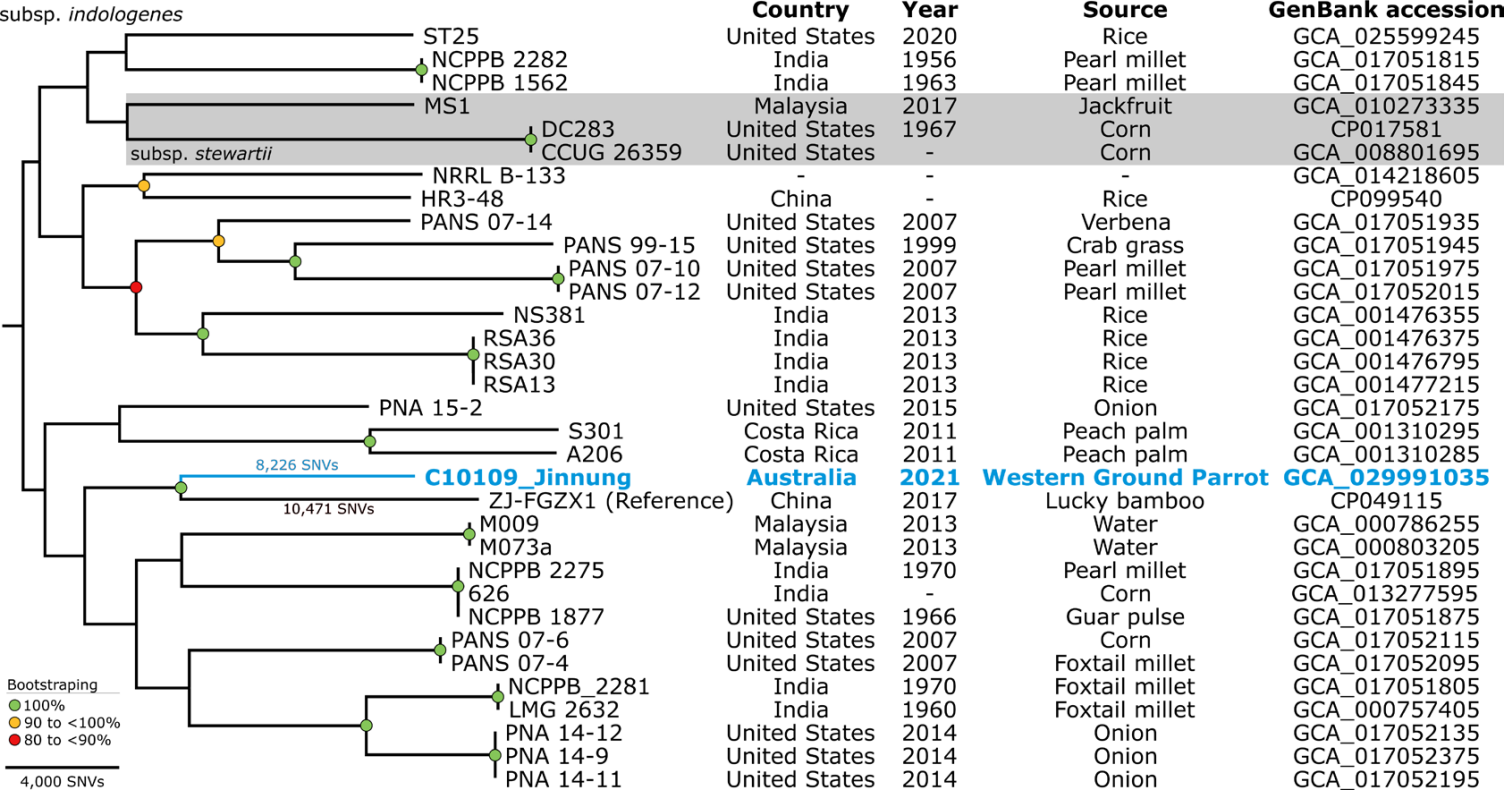
Maximum-parsimony phylogeny of *Pantoea stewartii *. The phylogeny was inferred from 100 720 core-genome single-nucleotide variants (SNVs) from 33 genomes and rooted at the midpoint. SNVs were derived from a core-genome alignment of 3 433 126 bp and are called against the reference chromosome of *P. stewartii* subsp. *indologenes* strain ZJ-FGZX1 (GenBank: CP049115). The blue taxon represents the strain sequenced in this report. Bootstrap values (using 1000 replicates) are shown. The consistency index is 0.44.

In this study, the genomic diversity of the draft genome sequence of C10109_Jinnung, obtained from an Australian Critically Endangered psittacine, was assessed by comparing it with 29 draft and three complete publicly available *P. stewartii* genomes (Fig. 3). Excluding the newly sequenced C10109_Jinnung obtained from an Australian Critically Endangered psittacine, the dataset comprises genomes of *P. stewartii* strains that have been identified from various sources, namely food (*n*=26), plants (*n*=3) and the environment (*n*=2), with one genome having an unknown source. Furthermore, the dataset consisted of *P. stewartii* genomes that were obtained from different geographical locations, including the USA (*n*=14), India (*n*=10), Malaysia (*n*=3), China (*n*=2) and Costa Rica (*n*=2), with two genomes having an unknown origin. The genomes in this collection were obtained over a period ranging from 1956 to 2020.

The alignment of 100 720 core-genome SNVs from 33 *P *. *stewartii* genomes was used to construct a maximum-parsimony phylogenetic tree (Fig. 3). However, the low consistency index of 0.44 indicates that there could be incongruence or homoplasy in the dataset, potentially affecting the accuracy of the inferred relationships. To address this, 1000 bootstrapped replicates were generated to increase confidence in the inferred relationships. Although the deeper branching patterns in the *P. stewartii* phylogeny remain more uncertain, the higher bootstrap values for more recent nodes suggest greater reliability in the inferred relationships between more ‘closely’ related taxa. However, the results of the phylogenetic analysis revealed that the 33 genomes exhibited a remarkable genomic diversity within the *P. stewartii* phylogeny. For example, the terminal branch leading to strain C10109_Jinnung is defined by 8226 SNVs, as determined by pairwise SNV distances, relative to the reference chromosome ZJ-FGZX1. Additionally, C10109_Jinnung was most genomically ‘similar’ to strain ZJ-FGZX1, which was isolated from lucky bamboo (*Dracaena sanderiana*) in China in 2017 [16]. However, this should be interpreted cautiously because C10109_Jinnung was separated from ZJ-FGZX1 by a pairwise SNV distance of 18 697 SNVs.

While C10109_Jinnung shares the same branch with strain ZJ-FGZX1 (subsp. *indologenes*), this does not necessarily contradict the possibility of it respresenting subsp. *stewartii*. To address this, we conducted an *in silico* analysis of the *galE* (encoding UDP-glucose 4-epimerase) and *recA* (encoding protein RecA) genes in the sequence of C10109_Jinnung. We then compared these genes with those of subsp. *stewartii* (DC283) and subsp. *indologenes* (ZJ-FGZX1) (Fig. S3). Based on *in silico* PCR and sequence alignments of the *recA* and *galE* genes from the complete DC283 and ZJ-FGZX1 genomes, it appears that C10109_Jinnung shares the same SNVs with ZJ-FGZX1 in most cases. However, one exception is an SNV C246T in the *recA* gene compared to ZJ-FGZX1 (matching subsp. *stewartii* DC283). This evidence suggests that C10109_Jinnung is probably a member of subsp. *indologenes*.

### Metagenomic diet

**Fig. 4.**
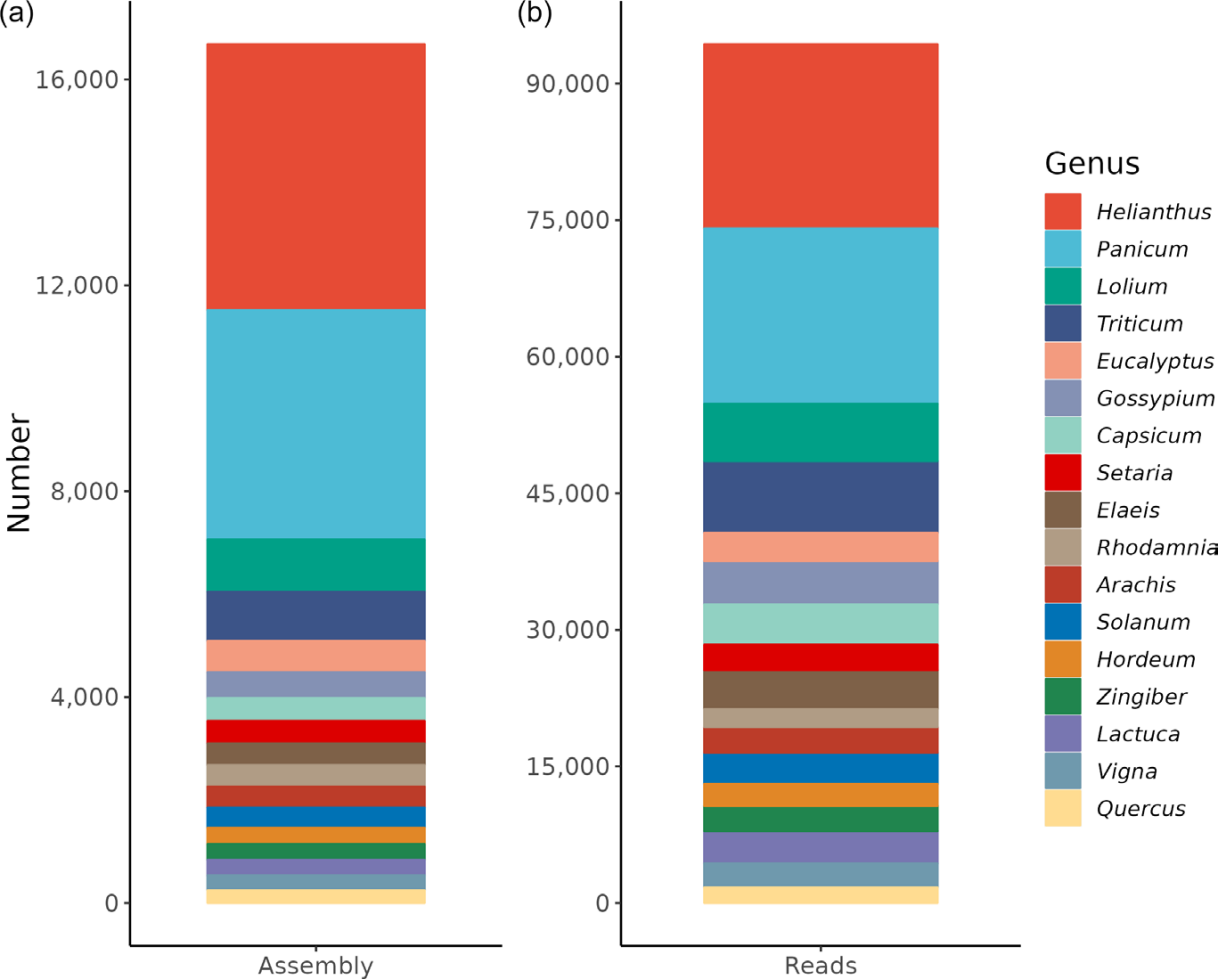
Potential *Pantoea stewartii* host genera. (**a**) Number of contigs in meta-assembly; (**b**) number of unassembled reads. Genera with over 1 % abundance are shown.

Taxonomic analysis using Kraken was conducted to investigate the composition in the C10109_Jinnung faecal sample. Analysis revealed a diverse range of taxa from part of the parrot’s diet. Metagenomic analysis revealed that the plant genera detected comprised mainly sunflowers (*Helianthus* spp.), panicgrass (*Panicum* spp.), ryegrass (*Lolium* spp.), eucalyptus (*Eucalyptus* spp.)*, Rhodamnia* spp., foxtail millet (*Setaria* spp.) and wheat (*Triticum* spp.). The species designation for the contigs were the common sunflower (*Helianthus annus*), Hall’s panicgrass (*Panicum hallii*), switchgrass (*Panicum virgatum*), flooded gum tree (*Eucalyptus grandis*), white myrtle (*Rhodamnia argentea*), millet (*Setaria italica*), annual ryegrass (*Lolium rigidum*) and common wheat (*Triticum aestivum*) (Fig. 4).

### Identification of a putative plasmid with cloning vector features

During the *de novo* assembly process of the sequence data for C10109_Jinnung, a putative plasmid with a length of 5839 bp was identified (named pC10109_Jinnung). This putative plasmid was sequenced to a coverage of approximately 1340-fold, indicating a potentially substantial presence within the faecal sample. The significance of this finding is currently unclear, and contamination cannot currently be ruled out. The sequence of the pC10109_Jinnung plasmid displays typical characteristics of a cloning vector, including T7 promoters and a multi-cloning site featuring seven restriction enzymes (Fig. S4). Detailed analysis and supplementary information regarding the putative plasmid pC10109_Jinnung, encompassing gene annotations, sequence similarities and distinctive features, can be found in the online supporting materials.

## Discussion

There have been no reported instances of *P. stewartii* strains being found from a bird source (to our knowledge). Despite the substantial economic losses incurred due to the impact of *P. stewartii* [5–7], with many countries classifying it as a quarantine organism [9], more global genomic data are needed for this bacterium. This study introduces a draft genome of a *P. stewartii* strain (C10109_Jinnung) obtained from Australia’s Critically Endangered western ground parrot/kyloring. It is important to clarify that our study is based on an exceptional occurrence that came about unexpectedly through our collaboration with Perth Zoo, focusing on a translocation project for the endangered western ground parrot/kyloring population in Western Australia. The bird in question, unfit for release into the wild, was sent to Perth Zoo for evaluation. This research does not encompass the relationship between *P. stewartii* and western ground parrots/kylorings across Western Australia. Rather, we present the first documented instance of *P. stewartii* in a bird source, supported by genomic analysis.

Genome analysis revealed the presence of multiple genetic elements within the chromosome, some associated with virulence. These included the type VI secretion system (cellular aggression mechanism [55]), flagella (mobility [56]), chromobacterium haem utilization and aerobactin operons (iron acquisition [57, 58]), as well as a P fimbriae operon (bacterial adhesion [59]). Unfortunately, there was insufficient DNA to perform long-read sequencing [60], and benefit from the improvements in draft assembly and resolving genomic complexity that this would have provided. We are exploring using Oxford Nanopore Technologies (long-read sequencing) with additional samples. Although the currently available genomes of *P. stewartii* display genetic diversity in the core genome, they present an underrepresented population. Moreover, this study shows that the genome of C10109_Jinnung is genomically distinct from other *P. stewartii* genomes.

### Insights from phylogenomic analysis and the call for broader genomic exploration

Previous studies have explored potential insect vectors of *P. stewartii *, but only in controlled environments [10–12]. The corn flea beetle stands out as an efficient vector due to its behaviour of depositing faecal matter directly on the leaf wounds it creates while feeding on the plant [13]. Therefore, unlike the corn flea beetle, the deposition of bird faecal matter on plants seems unlikely to cause infection in healthy plants unless there are pre-existing wounds. This is an important observation, in particular concerning the biosecurity risks associated with *P. stewartii *, but must be viewed in the context of the limitation that this study only investigated one genome from a *P. stewartii* strain obtained from a western ground parrot/kyloring. Although we acknowledge the importance of a larger sample size, practical challenges hindered our efforts. Restricted access to critically endangered parrot species in Australia and financial constraints prevented us from obtaining additional samples from various locations. Despite these challenges, publishing our findings establishes a groundwork for future sampling endeavours and funding requests. We eagerly encourage and anticipate that upcoming research will build upon our discoveries, fostering further exploration in this field. Therefore, additional research is necessary to investigate the role of pre-existing wounds in the transmission of plant infections through bird faecal matter and to sequence more *P. stewartii* genomes.

The true weight of our research emerged when we conducted phylogenetic analysis, revealing the evolutionary relationships and genetic context of C10109_Jinnung, which is probably classified as *P. stewartii* subsp. *indologenes* (Fig. 3). This study represents the most extensive phylogenomic investigation into *P. stewartii* (to our knowledge), encompassing a comprehensive framework with 32 additional genomes from diverse sources and locations. However, it is worth noting that while insightful, these 33 genomes offer a limited representation of the broader *P. stewartii* population. Nevertheless, this study has led to a better understanding of the genetic diversity and evolutionary dynamics within the species *P. stewartii *. Additionally, utilizing genomic DNA extracted directly from faecal material, rather than relying on cultured isolates, demonstrated the potential of culture-independent whole-genome sequencing for genotypic *P. stewartii* subspecies typing. This approach bypasses the limitations of traditional culturing methods and allows for the direct analysis of environmental samples. Future studies may also benefit from incorporating phenotypic strain culturing and biochemical tests for definitive subspecies identification [19]. These complementary techniques enable the isolation and characterization of specific strains, providing additional phenotypic information to enhance subspecies identification efforts.

This study’s core-genome SNV-based phylogenetic analysis revealed that C10109_Jinnung shared a most recent common ancestor with strain ZJ-FGZX1 (also subsp. *indologenes*), isolated from lucky bamboo in China in 2017 [16]. However, caution must be exercised when interpreting this finding, as C10109_Jinnung and ZJ-FGZX1 exhibit a substantial genetic divergence with an 18 697 pairwise SNV distance. The genetic variation and independent changes observed between C10109_Jinnung and ZJ-FGZX1 show substantial gaps in available *P. stewartii* genomes. Moreover, detecting *P. stewartii* in a bird host (albeit a single strain in a single host) prompts further investigation into its prevalence among other bird species in Western Australia. If additional instances of *P. stewartii* are discovered in Western Australia, replicating the current study would provide insights into the similarities or differences between any new findings and C10109_Jinnung. A broader analysis incorporating a more comprehensive range of *P. stewartii* strains and more extensive genomic datasets will provide a deeper understanding of the evolutionary relationships and population dynamics of *P. stewartii *.

### C10109_Jinnung’s virulence toolkit: flagella, fimbriae and iron acquisition systems

The availability of complete reference genomes played a crucial role in our study, enabling the identification of virulence factors in the draft genome of C10109_Jinnung, including flagella, fimbriae and siderophore/iron acquisition systems. These characteristics are not exclusive to C10109_Jinnung and are commonly shared among other *P. stewartii* genomes (Fig. 2). *P. stewartii* demonstrates impressive agar surface motility facilitated by flagella-dependent movement [61], and comparative analysis of complete genomes suggests the conservation of flagella-encoding genes among genomically distinct different *P. stewartii* strains (Fig. 2). The presence of P fimbriae genes in *P. stewartii* also raises intriguing possibilities for virulence, where P fimbriae can promote adherence to host tissues, promote colonization, and potentially contribute to urinary tract infections [62]. Moreover, genes encoding siderophore/iron acquisition systems in *P. stewartii* indicate an ability to compete for iron resources [63]. Consequently, the expression of flagella, P fimbriae and iron acquisition systems in *P. stewartii* may suggest a role in facilitating the adhesion to, and colonization of, host tissues, potentially enhancing its pathogenic potential and capacity to initiate infections and endure over time [64]. However, it is essential to acknowledge that these observations are based on a limited dataset, as only three complete genomes are currently accessible in the public domain [16–18].

### What about the expression vector?

Plasmids play a crucial role in molecular cloning, and various cloning vectors have been developed to facilitate the identification of plasmids with DNA inserts [65]. The Tet repressor (encoded by *tetR*) and specific aminophosphotransferases, such as *aph*(3′)-Ia and *aph*(3′)-IIa, have been widely used as selectable markers for tetracycline resistance [66–68] and kanamycin resistance in prokaryotic vectors, respectively [65]. The presence of pC10109_Jinnung, which shares similarities with an *Escherichia coli* expression vector and contains common *E. coli* protein expression vector sequences, suggests the potential for horizontal gene transfer [69]. Further investigation is needed to determine the origin of the possible horizontal gene transfer leading to the presence of the expression vector in the genome sequence data for C10109_Jinnung, whether it occurred naturally or because of laboratory contamination during experimental procedures. Furthermore, the high sequence coverage of pC10109_Jinnung (approximately 1340-fold) indicates a likely high abundance in the faecal sample if it is not due to contamination.

### Insights into the potential role of *P. stewartii* in seed-feeding bird-mediated transmission and implications for host expansion

Taxonomic analyses unveiled the intricate composition of the C10109_Jinnung faecal sample extending beyond the identified *P. stewartii *. We identified multiple matches to plant species that form part of the parrot’s diet, which could mainly reflect the diet while in the zoo. Nevertheless, employing this analysis on non-captured (wild) birds has the potential to yield invaluable insights regarding their geographical distribution and dietary patterns. *P. stewartii* can exploit a diverse range of plant and insect hosts, utilizing the transportation of host plants (including seeds) and insect vectors from neighbouring countries [70]. This, alongside our observations, suggests that seed-feeding birds might act as reservoirs and facilitate the spread of the bacteria, potentially leading to host shifts. Moreover, the crops and guts of birds could serve as a reservoir for transmitting *P. stewartii* to previously uninfected species. Additionally, avian dispersal could contribute to spreading contaminated seeds, increasing the risk of pathogenic host expansion.

Of the eight plant genera with the most contigs, two have already been identified as hosts for *P. stewartii* (wheat and millet, further supporting the identification of C10109_Jinnung as representing *P. stewartii* subsp. *indologenes *) [22], while others may also become susceptible to infection. For instance, flooded gum trees (*Eucalyptus grandis*) are a significant forestry crop, and the closely related *Erwinia psidii* has been found to have undergone a host shift to infect Myrtaceae and *Eucalyptus* in South America [71]. The metagenomic diet analyses provides a potential snapshot of the western ground parrot/kyloring diet, but it is not comprehensive and may not pick up everything consumed. For instance, while the western ground parrot/kyloring was provided with corn (Fig. S1), none was detected in the metagenomics diet analysis (Fig. 4). Furthermore, the substantial implications of *P. stewartii* host expansion facilitated by birds on food security and economic productivity cannot be overstated.

## Conclusion

This study highlights the need for more comprehensive genomic data on *P. stewartii *, a bacterium causing significant economic losses and classified as a quarantine organism. While no previous reports exist of *P. stewartii* strains isolated from a bird source, the draft genome analysis of C10109_Jinnung from one of the Critically Endangered Australian parrots reveals genetic elements associated with virulence, including cellular aggression, mobility, iron acquisition and bacterial adhesion. However, it is important to acknowledge the limited representation of *P. stewartii* genomes in the current dataset, potentially missing intermediate evolutionary lineages. Future research should investigate the specific genetic changes and mechanisms driving this divergence, explore functional implications of genetic variations, and expand the analysis to encompass a broader range of *P. stewartii* strains and larger genomic datasets. Incorporating diverse strains from various sources and locations would enable a comprehensive exploration of the genomic landscape of *P. stewartii *, facilitating insights into the epidemiology, host interactions, and the potential for antibiotic resistance acquisition and dissemination. Additionally, identifying plant species in the C10109_Jinnung sample suggests potential hosts or carriers for *P. stewartii* spread, necessitating investigation into bird-mediated transmission and its implications for plant host expansion and food security.

## Supplementary Data

Supplementary material 1
