# Supplementary material for: A discovery down under: decoding the draft genome sequence of Pantoea stewartii from Australia’s Critically Endangered western ground parrot/kyloring (Pezoporus flaviventris)
Source: Microb Genom. 2023 Sep 4;9(9):001101. doi: 10.1099/mgen.0.001101 (PMC10569725; doi:10.1099/mgen.0.001101)
Supplement: Supplementary material 1 [file mgen-9-1101-s001.pdf]

SUPPLEMENTARY APPENDIX

A discovery down under: Decoding the draft genome sequence of *Pantoea stewartii* from Australia's Critically Endangered Western Ground Parrot/Kyloring (*Pezoporus flaviventris*)

Author names

Rhys T. White[1], William Taylor[2], Natalie Klukowski[3], Rebecca Vaughan-Higgins[4], Ernest Williams[5], Steve Petrovski[3], Jayson J. A. Rose[3], Subir Sarker[3,6,*]

Affiliation

[1]Institute of Environmental Science and Research, Wellington, New Zealand

[2]Institute of Environmental Science and Research, Christchurch, New Zealand

[3]La Trobe University, School of Agriculture, Biomedicine and Environment, Department of Microbiology, Anatomy, Physiology and Pharmacology, Melbourne, Victoria, Australia

[4]Perth Zoo, South Perth, Western Australia, Australia

[5]Institute of Environmental Science and Research, Wallaceville, New Zealand

[6]Biomedical Sciences & Molecular Biology, College of Public Health, Medical and Veterinary Sciences, James Cook University, Townsville, QLD, Australia.

Corresponding author

*Corresponding author: Telephone: +61-7-4781-4336; E-mail: subir.sarker@jcu.edu.au

Keywords

Pantoea stewartii subsp. *indologenes*; strain C10109_Jinnung; *Enterobacterales*; phylogenomics; birds; Australia

<u>This file includes:</u>

Supplementary Figure S1. Seed provisions and native food plants for the Critically Endangered Western Ground Parrot/Kyloring (*Pezoporus flaviventris*) (named C10109 Jinnung) during temporary holding.

Supplementary Figure S2. Genome sequence coverage plot for *Pantoea stewartii* strain C10109_Jinnung.

Supplementary Figure S3. Sequence alignments for the *rec*A and *gal*E genes from *Pantoea stewartii* subspecies *stewartii* (DC283) and *indologenes* (ZJ-FGZX1) genomes.

Supplementary Figure S4. Circularised model of putative expression vector pC10109_Jinnung isolated from *Pantoea stewartii* C10109_Jinnung.

Supplementary Table S1. Species identification based on Kraken2 analysis (read assignment).

Supplementary Table S2. Whole genome sequences of 32 global *Pantoea stewartii* strains used in this investigation.

Supplementary Table S3. Species identification based on Kraken2 analysis (meta-assembly assignment).

Supplementary Results

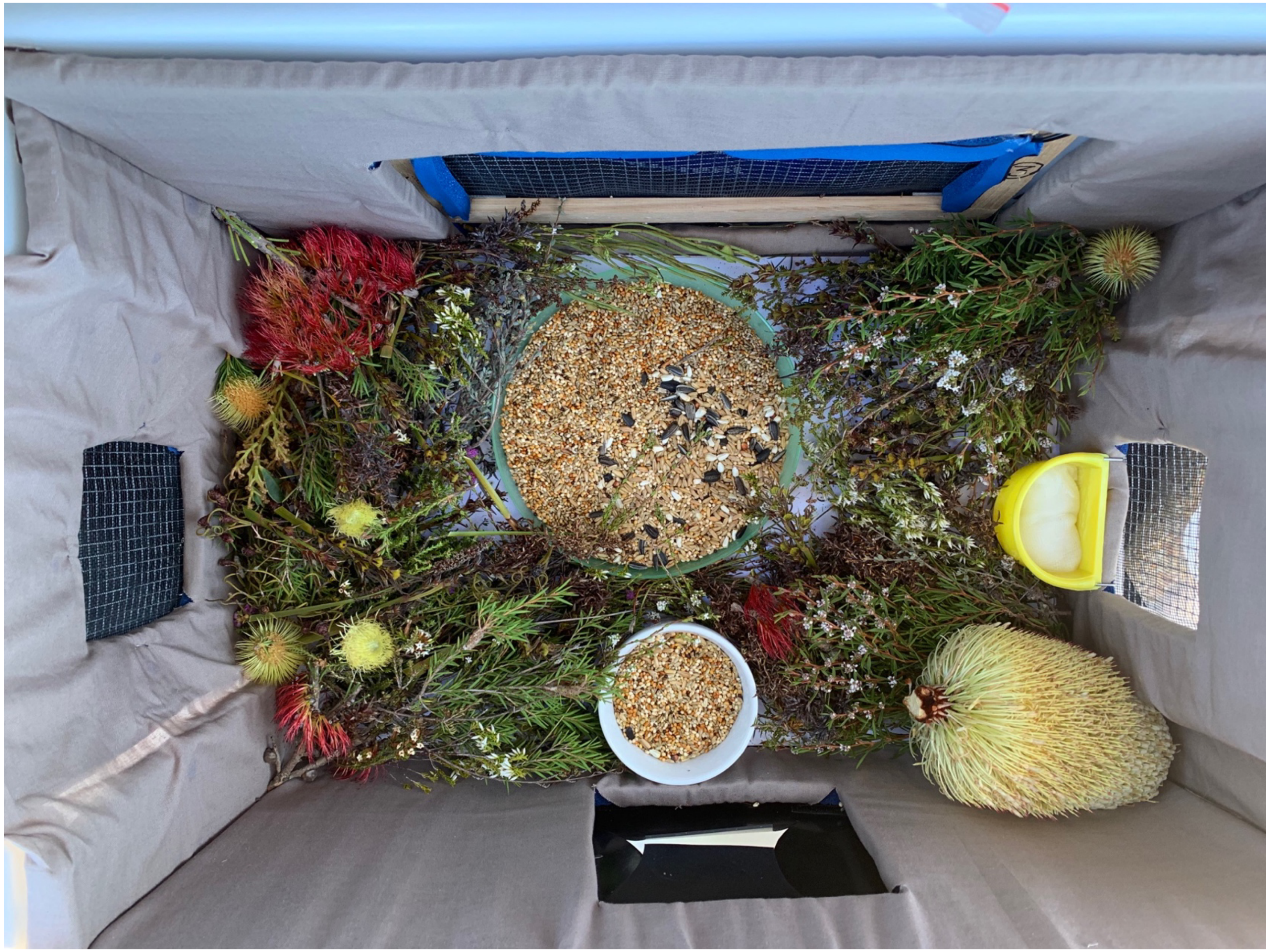

Supplementary Figure S1. Seed provisions and native food plants for the Critically Endangered Western Ground Parrot/Kyloring (*Pezoporus flaviventris*) (named C10109 Jinnung) during temporary holding. The seed mix provided to C10109 Jinnung during temporary holding, including grey-striped sunflower, black sunflower, safflower, de-hulled oats, canary seed, white millet, Japanese millet, red panicum, yellow panicum, and canola. Additionally, native food plants were provided to C10109 Jinnung during this period, ensuring a diet consistent with its natural food sources.

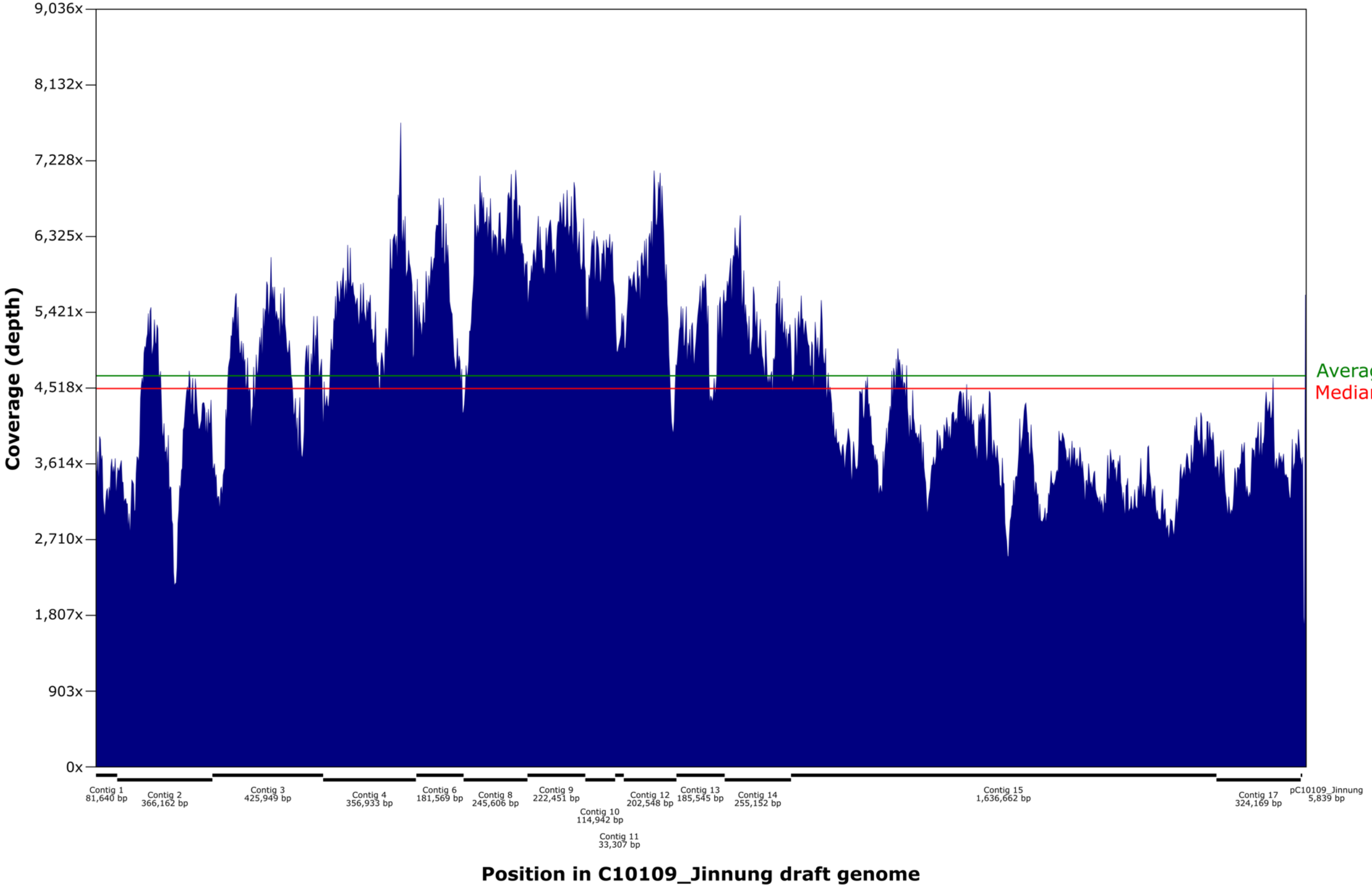

Supplementary Figure S2. Genome sequence coverage plot for *Pantoea stewartii* strain C10109_Jinnung. The plot was calculated with the script "wgscoverageplotter.jar" from the Java utilities for Bioinformatics (JVARKIT) (https://github.com/lindenb/jvarkit) (1). Contiguous sequences (contigs) smaller than 2,000 bp were excluded from visualisation. These contigs include: contig 5 (1,849 bp); contig 7 (552 bp); contig 16 (851 bp); contig 19 (1,958 bp); and contig 20 (1,507 bp).

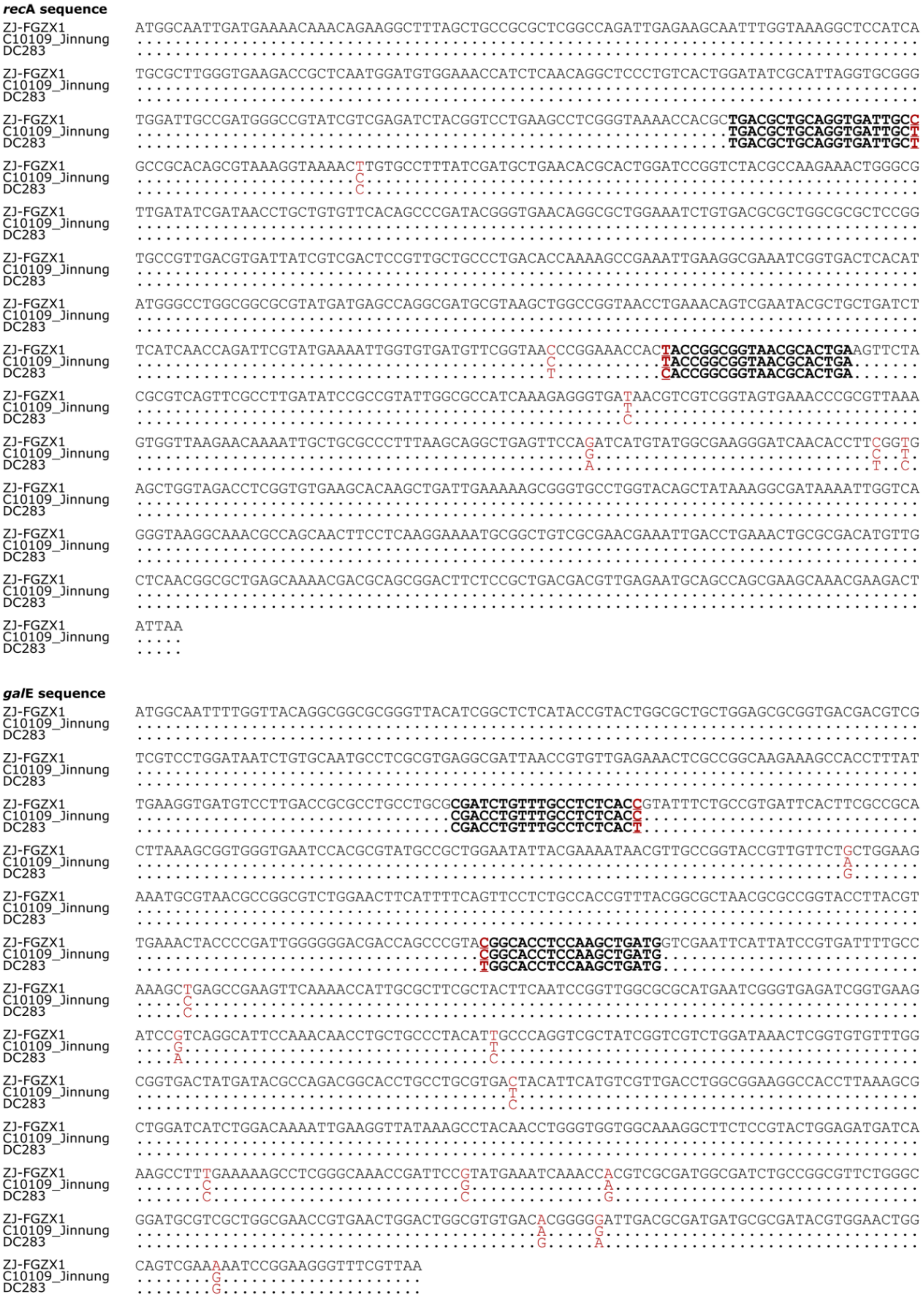

Supplementary Figure S3. Sequence alignments for the *rec*A and *gal*E genes from *Pantoea stewartii* subspecies *stewartii* (DC283) and *indologenes* (ZJ-FGZX1) genomes. Red nucleotides represent single-nucleotide variants (SNVs) identified when using the chromosome of *P. stewartii* subsp. *indologenes* strain ZJ-FGZX1 (GenBank: CP049115) as a reference sequence. Emboldened nucleotides represent specific primers for the differentiation of *P. stewartii* subsp. *indologenes* and *P. stewartii subsp. stewartii* by stepdown Polymerase Chain Reaction based on findings from Gehring *et al.* (2).

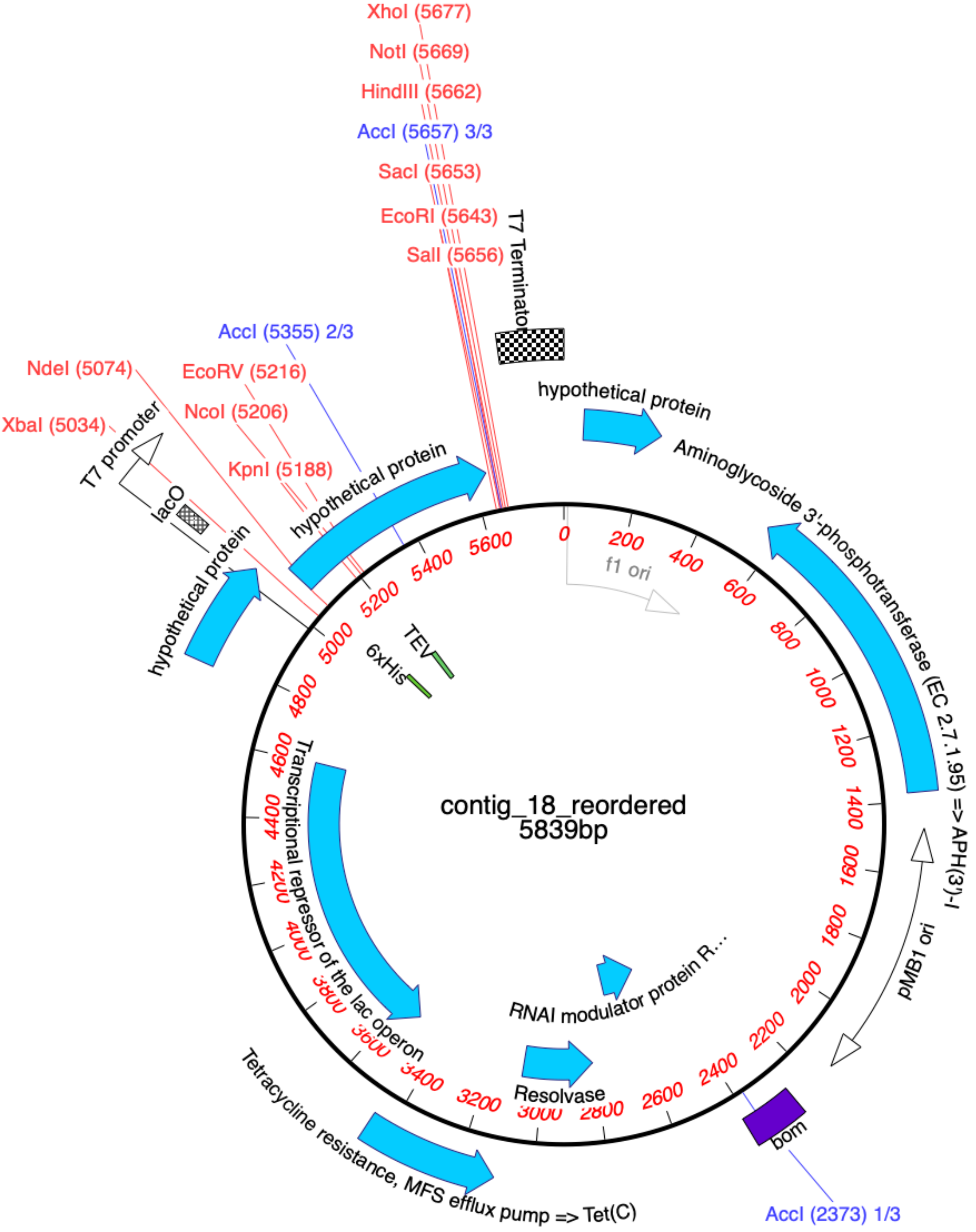

Supplementary Figure S4. Circularised model of putative expression vector pC10109_Jinnung isolated from *Pantoea stewartii* C10109_Jinnung. A circularised annotated map of the 5,839 base contig. Restriction sites are shown in red; open reading frames are shown as blue arrows; the origins of replication and promoters are shown as stick arrows; other features are shown as grey boxes.

Supplementary Table S1. Species identification based on Kraken2 analysis (read assignment)

Percentage of reads in clade/taxon	Number of reads in clade	Number of reads in clade but not further classified	Rank code	NCBI taxonomic ID	Scientific name
4.75	3854321	3854321	U	0	Unclassified
**90.85**	**73662008**	**47112566**	**S**	**66269**	***Pantoea stewartii***
1.05	849732	747779	S	553	*Pantoea ananatis*
0.16	127320	127320	S	59814	*Pantoea dispersa*
0.14	114910	114910	S	549	*Pantoea agglomerans*
0.09	76471	76471	S	2490851	*Pantoea* sp. CCBC3-3-1
0.01	4169	4169	S	592316	*Pantoea* sp. At-9b
0	2654	2654	S	2052056	*Pantoea* sp. MSR2
0	1147	1147	S	2575375	*Pantoea* sp. SO10
0.02	13868	13567	S	470934	*Pantoea vagans*
0	3402	3402	S	1891675	*Pantoea alhagi*
0	1902	1902	S	470933	*Pantoea eucalypti*
0	1508	1508	S	55209	*Pantoea cypripedii*
0.17	141680	141680	S	2872648	*Mixta* sp. X22927
0	2521	2521	S	55212	*Erwinia rhapontici*
0.15	120769	120769	S	2058152	*Klebsiella grimontii*
0.04	30651	30486	S	573	*Klebsiella pneumoniae*
0.02	14003	13987	S	244366	*Klebsiella variicola*
0.01	6742	6742	S	548	*Klebsiella aerogenes*
0	1960	1936	S	1463165	*Klebsiella quasipneumoniae*
0.02	14141	14141	S	54291	*Raoultella ornithinolytica*
0.02	17992	17992	S	299767	*Enterobacter ludwigii*
0.01	5976	4991	S	158836	*Enterobacter hormaechei*
0.01	5428	5428	S	1812935	*Enterobacter roggenkampii*
0.01	5303	5303	S	61645	*Enterobacter asburiae*
0.01	5070	5062	S	550	*Enterobacter cloacae*
0	3556	3556	S	69218	*Enterobacter cancerogenus*
0.01	9775	9775	S	539813	*Enterobacter mori*
0	1896	1896	S	2742656	*Enterobacter* sp. RHBSTW-00593
0	1744	1744	S	2831891	*Enterobacter* sp. JBIWA005
0	1392	1392	S	2831890	*Enterobacter* sp. JBIWA003
0.03	26728	26019	S	562	*Escherichia coli*
0.01	7749	7738	S	1158459	*Kosakonia sacchari*
0.01	6425	3851	S	208223	*Kosakonia cowanii*
0	3514	3513	S	283686	*Kosakonia radicincitans*
0	2722	2722	S	497725	*Kosakonia oryzae*
0	1342	1342	S	2492396	*Kosakonia* sp. CCTCC M2018092
0	1164	1164	S	551989	*Kosakonia arachidis*
0	2974	2974	S	2742657	*Citrobacter* sp. RHBSTW-00599
0.01	4525	4525	S	2546350	*Citrobacter arsenatis*
0	1912	1912	S	546	*Citrobacter freundii*
0.01	7106	3717	S	28901	*Salmonella enterica*
0	1709	1672	S	54736	*Salmonella bongori*
0.01	8256	8256	S	2714951	*Leclercia* sp. 29361
0.01	6790	6790	S	61646	*Lelliottia amnigena*
0	1576	1576	S	158823	*Cedecea lapagei*
0	1526	0	S	413497	*Cronobacter dublinensis*
0.01	11981	11981	S	29485	*Yersinia rohdei*
0.01	5049	5040	S	633	*Yersinia pseudotuberculosis*
0	1074	1074	S	29486	*Yersinia ruckeri*
0.01	4661	2031	S	82996	*Serratia plymuthica*
0	2555	2552	S	615	*Serratia marcescens*
0	2423	2423	S	61652	*Serratia rubidaea*
0	2027	2027	S	137545	*Serratia quinivorans*
0	1787	1755	S	34038	*Rahnella aquatilis*
0	1366	1366	S	1805933	*Rahnella* sp. ERMR1:05
0	2061	2061	S	1646377	*Rouxiella badensis*

Supplementary Table S1. Species identification based on Kraken2 analysis (…*continued*)

Percentage of reads in clade/taxon	Number of reads in clade	Number of reads in clade but not further classified	Rank code	NCBI taxonomic ID	Scientific name
0	1625	1625	S	41202	*Ewingella americana*
0.02	19447	18	S	40576	*Xenorhabdus bovienii*
0	2779	2778	S	584	*Proteus mirabilis*
0	1013	20	S	554	*Pectobacterium carotovorum*
0	2617	2617	S	1109412	*Brenneria goodwinii*
0	2609	2609	S	1239307	*Sodalis praecaptivus*
0.01	4872	4872	S	47885	*Pseudomonas oryzihabitans*
0	1521	1521	S	237610	*Pseudomonas psychrotolerans*
0	2569	2569	S	40269	*Aliivibrio salmonicida*
0	1790	1790	S	2871174	*Stenotrophomonas* sp. DR822
0	2149	0	S	271097	*Shewanella sediminis*
0	2697	2697	S	475937	*Rhodopseudomonas boonkerdii*
0.01	10067	10062	S	88688	*Caulobacter segnis*
0	3145	3143	S	155892	*Caulobacter vibrioides*
0	2276	2276	S	69665	*Caulobacter* sp. FWC26
0	1079	1079	S	2482763	*Sphingopyxis* sp. YF1
0.01	7206	7206	S	1707785	*Massilia* sp. WG5
0	1736	1736	S	2769491	*Massilia* sp. LPB0304
0	1148	1148	S	2861282	*Massilia* sp. NP310
0.01	7371	7371	S	2728020	*Massilia forsythiae*
0	3370	3370	S	1141883	*Massilia putida*
0	1576	1576	S	2753607	*Rhizobacter* sp. AJA081-3
0	1569	966	S	2047	*Rothia dentocariosa*
0	1031	1011	S	1747	*Cutibacterium acnes*
0	2025	2025	S	59893	*Paenibacillus peoriae*
0	1170	1170	S	2675878	*Hymenobacter* sp. BRD128
0.24	196462	196462	S	9606	*Homo sapiens*
0	1113	1113	S	3914	*Vigna angularis*
0	1137	1137	S	3880	*Medicago truncatula*
0	1120	1120	S	3827	*Cicer arietinum*
0	1665	1665	S	3871	*Lupinus angustifolius*
0	2143	2143	S	3988	*Ricinus communis*
0	1211	1211	S	3986	*Mercurialis annua*
0	1253	1253	S	32201	*Carya illinoinensis*
0	1540	1540	S	102211	*Benincasa hispida*
0	3062	3062	S	71139	*Eucalyptus grandis*
0	1969	1969	S	178133	*Rhodamnia argentea*
0	1105	1105	S	22663	*Punica granatum*
0	1400	1400	S	3635	*Gossypium hirsutum*
0	1102	1102	S	90675	*Camelina sativa*
0.02	20240	20240	S	4232	*Helianthus annuus*
0	3575	3575	S	4236	*Lactuca sativa*
0	1253	0	S	4039	*Daucus carota*
0.01	4439	4439	S	4072	*Capsicum annuum*
0	1804	1804	S	172797	*Solanum stenotomum*
0	2094	2094	S	49451	*Nicotiana attenuata*
0	1567	0	S	4146	*Olea europaea*
0	1041	1041	S	253017	*Impatiens glandulifera*
0.01	11239	11239	S	206008	*Panicum hallii*
0.01	7442	7442	S	38727	*Panicum virgatum*
0	1060	1060	S	4555	*Setaria italica*
0	1610	1610	S	4577	*Zea mays*
0	1054	1054	S	4558	*Sorghum bicolor*
0	1945	1945	S	4565	*Triticum aestivum*
0	1306	1306	S	85692	*Triticum dicoccoides*
0	2380	0	S	4513	*Hordeum vulgare*
0.01	6296	6296	S	89674	*Lolium rigidum*

Supplementary Table S1. Species identification based on Kraken2 analysis (…*continued*)

Percentage of reads in clade/taxon	Number of reads in clade	Number of reads in clade but not further classified	Rank code	NCBI taxonomic ID	Scientific name
0	1260	1260	S	4615	*Ananas comosus*
0.01	4283	4283	S	51953	*Elaeis guineensis*
0	1145	1145	S	42345	*Phoenix dactylifera*
0	2768	2768	S	94328	*Zingiber officinale*
0	1918	1918	S	4686	*Asparagus officinalis*
0	2617	2617	S	54955	*Telopea speciosissima*
0	1031	1031	S	60698	*Macadamia integrifolia*
0	1998	1998	S	3469	*Papaver somniferum*
0	1336	0	S	5850	*Plasmodium knowlesi*

Displaying only "Species" rank with ≥1000 classified reads

Supplementary Table S2. Whole genome sequences of 32 global *Pantoea stewartii* strains used in this investigation

Strain	Subspecies	Country	Collection date	Source	Bio Project ID	BioSample	GenBank	PubMed ID
ST25	-	United States	2020	Rice	PRJNA880563	SAMN30852752	GCA_025599245	-
NCPPB 2282	*indologenes*	India	1956	Pearl millet	PRJNA676043	SAMN16866627	GCA_017051815	33599528
NCPPB 1562	*indologenes*	India	1963	Pearl millet	PRJNA676043	SAMN16866628	GCA_017051845	33599528
MS1	*stewartii*	Malaysia	2017	Jackfruit	PRJNA521843	SAMN10915378	GCA_010273335	-
DC283	*stewartii*	United States	1967	Corn	PRJNA342501	SAMN05761485	CP017581	28572317
CCUG 26359	*stewartii*	United States	-	Corn	PRJNA563568	SAMN12697580	GCA_008801695	-
NRRL B-133	-	-	-	-	PRJNA646986	SAMN15572496	GCA_014218605	-
HR3-48	-	China	-	Rice	PRJNA844595	SAMN28835860	CP099540	36482381
PANS 07-14	*indologenes*	United States	2007	Verbene	PRJNA676043	SAMN16866623	GCA_017051935	33599528
PANS 99-15	*indologenes*	United States	1999	Crab grass	PRJNA676043	SAMN16866624	GCA_017051945	33599528
PANS 07-10	*indologenes*	United States	2007	Pearl millet	PRJNA676043	SAMN16866621	GCA_017051975	33599528
PANS 07-12	*indologenes*	United States	2007	Pearl millet	PRJNA676043	SAMN16866622	GCA_017052015	33599528
NS381	-	India	2013	Rice	PRJNA278247	SAMN03401412	GCA_001476355	26793183
RSA36	-	India	2013	Rice	PRJNA278253	SAMN03401418	GCA_001476375	26793183
RSA30	-	India	2013	Rice	PRJNA278250	SAMN03401415	GCA_001476795	26793183
RSA13	-	India	2013	Rice	PRJNA278249	SAMN03401414	GCA_001477215	26793183
PNA 15-2	*indologenes*	United States	2015	Onion	PRJNA676043	SAMN16866618	GCA_017052175	33599528
S301	-	Costa Rica	2011	Peach palm	PRJNA292332	SAMN03968918	GCA_001310295	-
A206	-	Costa Rica	2011	Peach palm	PRJNA292338	SAMN03968921	GCA_001310285	-
ZJ-FGZX1	*indologenes*	China	2017	Lucky bamboo	PRJNA607905	SAMN14150199	CP049115	-
M009	-	Malaysia	2013	Water	PRJNA263996	SAMN03112721	GCA_000786255	25635007
M073a	-	Malaysia	2013	Water	PRJNA265925	SAMN03154073	GCA_000803205	25700398
NCPPB 2275	*indologenes*	India	1970	Pearl millet	PRJNA676043	SAMN16866625	GCA_017051895	33599528
626	-	India	-	Corn	PRJNA322085	SAMN05013688	GCA_013277595	-
NCPPB 1877	*indologenes*	United States	1966	Guar pulse	PRJNA676043	SAMN16866626	GCA_017051875	33599528
PANS 07-6	*indologenes*	United States	2007	Corn	PRJNA676043	SAMN16866620	GCA_017052115	33599528
PANS 07-4	*indologenes*	United States	2007	Foxtail millet	PRJNA676043	SAMN16866619	GCA_017052095	33599528
NCPPB_2281	*indologenes*	India	1970	Foxtail millet	PRJNA676043	SAMN16866629	GCA_017051805	33599528
LMG 2632	*indologenes*	India	1960	Foxtail millet	PRJNA252992	SAMN02905159	GCA_000757405	-
PNA 14-12	*indologenes*	United States	2014	Onion	PRJNA676043	SAMN16866617	GCA_017052135	33599528
PNA 14-9	*indologenes*	United States	2014	Onion	PRJNA676043	SAMN16866615	GCA_017052375	33599528
PNA 14-11	*indologenes*	United States	2014	Onion	PRJNA676043	SAMN16866616	GCA_017052195	33599528

Supplementary Table S3. Species identification based on Kraken2 analysis (meta-assembly assignment)

Percentage of reads in clade/taxon	Number of reads in clade	Number of reads in clade but not further classified	Rank code	NCBI taxonomic ID	Scientific name
82.01	717194	717194	U	0	Unclassified
0.7	6094	6094	S	549	*Pantoea agglomerans*
0.61	5294	5294	S	59814	*Pantoea dispersa*
0.03	273	206	S	470934	*Pantoea vagans*
0.01	106	59	S	66269	*Pantoea stewartii*
0.2	1781	1006	S	208223	*Kosakonia cowanii*
0.18	1577	1503	S	562	*Escherichia coli*
0.19	1636	1636	S	47885	*Pseudomonas oryzihabitans*
0.02	136	125	S	303	*Pseudomonas putida*
0.06	497	497	S	237610	*Pseudomonas psychrotolerans*
0.01	130	93	S	294	*Pseudomonas fluorescens*
0.01	106	87	S	729	*Haemophilus parainfluenzae*
0.01	102	102	S	2603276	*Methylobacterium* sp. WL1
0.02	143	143	S	374432	*Methylobacterium tardum*
0.01	111	111	S	270351	*Methylobacterium aquaticum*
0.01	100	100	S	269660	*Methylobacterium brachiatum*
0.33	2880	2879	S	88688	*Caulobacter segnis*
0.09	783	781	S	155892	*Caulobacter vibrioides*
0.06	538	538	S	69665	*Caulobacter* sp. FWC26
0.02	182	182	S	2708539	*Caulobacter soli*
0.02	164	164	S	2010972	*Caulobacter rhizosphaerae*
0.02	160	160	S	1679497	*Caulobacter flavus*
0.02	138	138	S	2653203	*Sphingomonas* sp. CL5.1
0.01	102	102	S	2219696	*Sphingomonas* sp. FARSPH
0.01	127	127	S	1549858	*Sphingomonas taxi*
0.01	105	105	S	1560345	*Sphingomonas panacis*
0.01	131	131	S	1484109	*Lichenicola cladoniae*
0.01	100	100	S	504468	*Rhodovastum atsumiense*
0.27	2334	2334	S	1707785	*Massilia* sp. WG5
0.06	497	497	S	2769491	*Massilia* sp. LPB0304
0.04	349	349	S	2861282	*Massilia* sp. NP310
0.02	139	139	S	2852099	*Massilia* sp. HC52
0.01	112	112	S	2861283	*Massilia* sp. PAMC28688
0.26	2316	2316	S	2728020	*Massilia forsythiae*
0.11	972	972	S	1141883	*Massilia putida*
0.02	199	199	S	945844	*Massilia oculi*
0.02	147	147	S	2045208	*Massilia violaceinigra*
0.04	316	316	S	1071679	*Caballeronia grimmiae*
0.02	136	136	S	1296669	*Aquabacterium olei*
0.02	149	149	S	1561023	*Curtobacterium* sp. MR_MD2014
0.01	131	0	S	2035	*Curtobacterium flaccumfaciens*
0.06	498	299	S	2047	*Rothia dentocariosa*
0.01	120	96	S	43675	*Rothia mucilaginosa*
0.04	362	355	S	1747	*Cutibacterium acnes*
0.03	305	195	S	544580	*Actinomyces oris*
0.02	173	173	S	2748863	*Paenibacillus* sp. E222
0.02	159	159	S	59893	*Paenibacillus peoriae*
0.02	135	135	S	2912824	*Niallia* sp. Man26
0.03	247	198	S	28037	*Streptococcus mitis*
0.01	117	80	S	1303	*Streptococcus oralis*
0.01	105	76	S	29466	*Veillonella parvula*
4.86	42497	42497	S	9606	*Homo sapiens*
0.01	125	125	S	69781	*Penicillium oxalicum*
0.59	5172	5172	S	4232	*Helianthus annuus*
0.03	298	298	S	4236	*Lactuca sativa*
0.01	117	0	S	4265	*Cynara cardunculus*

Supplementary Table S3. Species identification based on Kraken2 analysis (…*continued*)

Percentage of reads in clade/taxon	Number of reads in clade	Number of reads in clade but not further classified	Rank code	NCBI taxonomic ID	Scientific name
0.05	441	441	S	4072	*Capsicum annuum*
0.01	119	119	S	4081	*Solanum lycopersicum*
0.01	108	108	S	28526	*Solanum pennellii*
0.02	156	156	S	172797	*Solanum stenotomum*
0.02	192	192	S	49451	*Nicotiana attenuata*
0.01	102	102	S	35885	*Ipomoea triloba*
0.02	186	0	S	4146	*Olea europaea*
0.01	106	106	S	180675	*Salvia splendens*
0.02	138	138	S	253017	*Impatiens glandulifera*
0.01	122	122	S	3821	*Cajanus cajan*
0.01	120	120	S	3885	*Phaseolus vulgaris*
0.01	107	107	S	3880	*Medicago truncatula*
0.01	107	107	S	57577	*Trifolium pratense*
0.01	120	120	S	3827	*Cicer arietinum*
0.02	157	157	S	3871	*Lupinus angustifolius*
0.02	141	141	S	3986	*Mercurialis annua*
0.02	181	181	S	102211	*Benincasa hispida*
0.07	608	608	S	71139	*Eucalyptus grandis*
0.05	420	420	S	178133	*Rhodamnia argentea*
0.02	143	143	S	3635	*Gossypium hirsutum*
0.35	3037	3037	S	206008	*Panicum hallii*
0.15	1301	1301	S	38727	*Panicum virgatum*
0.02	163	163	S	4555	*Setaria italica*
0.01	124	124	S	4556	*Setaria viridis*
0.02	190	190	S	4577	*Zea mays*
0.02	132	132	S	4558	*Sorghum bicolor*
0.03	244	244	S	4565	*Triticum aestivum*
0.02	168	168	S	85692	*Triticum dicoccoides*
0.01	107	107	S	4572	*Triticum urartu*
0.04	316	0	S	4513	*Hordeum vulgare*
0.12	1013	1013	S	89674	*Lolium rigidum*
0.01	105	105	S	4615	*Ananas comosus*
0.05	424	424	S	51953	*Elaeis guineensis*
0.02	142	142	S	42345	*Phoenix dactylifera*
0.04	308	308	S	94328	*Zingiber officinale*
0.01	109	0	S	4641	*Musa acuminata*
0.02	155	155	S	4686	*Asparagus officinalis*
0.01	119	0	S	29710	*Dioscorea cayenensis*
0.02	168	168	S	54955	*Telopea speciosissima*
0.01	120	120	S	60698	*Macadamia integrifolia*
0.02	209	209	S	3469	*Papaver somniferum*
0.03	281	281	S	2059380	Flamingopox virus FGPVKD09

Displaying only "Species" rank with ≥100 classified reads

Supplementary Results

During the *de novo* assembly process of the sequence data for C10109_Jinnung, a putative plasmid with a length of 5,839 bp was identified (named pC10109_Jinnung). This putative plasmid was sequenced to a coverage of approximately 1,340-fold, indicating a potentially substantial presence within the faecal sample. The GC percentage of the plasmid is 53.64%, which is within the typical range for bacterial DNA. BLASTn comparison revealed that pC10109_Jinnung showed similarity to the complete sequence of the expression vector p864INS (GenBank: AF116269), isolated from *Escherichia coli*, with 92% query coverage and 100% nucleotide identity. Furthermore, the circularised putative plasmid contains several sequences commonly found in protein expression vectors for use in *E. coli* (Supplementary Materials, Figure S4). Gene annotations showed several open reading frames found in expression vectors such as the *aph*(3′)-Ia (Aminoglycoside 3′-phosphotransferase) and genes lac operon repressor that all had 100% identity to *E. coli* according to nucleotide-protein BLAST (BLASTx) against the NCBI non-redundant database as well as the *tetR* gene fragment that is 91% identical to *E. coli*. However, the genes annotated as "hypothetical protein" were located within a multi-cloning site characterised by multiple unique tandem restriction sites, including NcoI and NdeI that produce an "AUG" start codon. A T7 promoter and a T7 terminator, used to initiate and terminate mRNA transcription respectively in DE3 lysogen-containing *E. coli* expression strains were also present. A "6X HIS tag", used for Nickel based protein purification, and the Tobacco Etch Virus (TEV), used to cleave off protein purification tags, were also present. The "hypothetical protein" open reading frame adjacent to the multi-cloning site and flanked by the T7 promoter and terminator was 100% identical to a Psittacine adenovirus 2 core protein sequence using BLASTx and the NCBI non-redundant database. Other features include the pMB1 *E. coli*-derived and f1 phage-derived origins of replication. Notably, the genome sequence analysed from pC10109_Jinnung did not exhibit high similarity to sequences from *P. stewartii* according to the BLASTx analysis.

References

1. **Lindenbaum P.** JVarkit: java-based utilities for Bioinformatics. Figshare doi: 10.6084/m9.figshare.1425030

2. **Gehring I, Wensing A, Gernold M, Wiedemann W, Coplin DL, Geider K**. Molecular differentiation of *Pantoea stewartii* subsp. *indologenes* from subspecies *stewartii* and identification of new isolates from maize seeds. *Journal of Applied Microbiology* 2014;116:1553-1562 doi: 10.1111/jam.12467
